# Isolation and characterization of extracellular vesicles from EGFR mutated lung cancer cells

**DOI:** 10.1007/s10238-025-01643-w

**Published:** 2025-04-10

**Authors:** Dian Jamel Salih, Katrin S. Reiners, Roberta Alfieri, Ahmed Mohammed Salih, Zulema Antonia Percario, Mariantonietta Di Stefano, Sollitto Francesco, Elisabetta Affabris, Gunther Hartmann, Teresa Santantonio

**Affiliations:** 1 Department of Medical and Surgical Sciences, University of Foggia, Via Napoli, 121, 71122 Foggia, Italy; 2 Department of Anatomy, Biology and Histology, College of Medicine, University of Duhok, Duhok, Iraq; 3 Institute of Clinical Chemistry and Clinical Pharmacology, University Hospital Bonn, Bonn, Germany; 4 Department of Medicine and Surgery, University of Parma, Parma, Italy; 5 College of Nursing, University of Duhok, Duhok, Iraq; 6 Department of Science, University Roma Tre, Rome, Italy

**Keywords:** EGFR, Extracellular vesicles, Lung cancer

## Abstract

The epidermal growth factor receptor (EGFR) signaling pathway is essential for cellular processes such as proliferation, survival, and migration. Dysregulation of EGFR signaling is frequently observed in non-small cell lung cancer (NSCLC) and is associated with poor prognosis. This study aims to isolate and characterize extracellular vesicles (EVs) released by mutant EGFR lung cancer cell line PC9 and compare them with wild-type EGFR lung cancer cell line A549, while also evaluating the effect of gefitinib treatment on EV secretion and cargo composition. The two lung cancer cell lines were cultured with 2% EV-free serum, and EVs were subsequently isolated by differential ultra centrifugation. EVs were characterized by nanoparticle tracking analysis (NTA) and transmission electron microscopy (TEM) for quantification size and shape determination. Western blot analysis confirmed the enrichment and purity of isolated EVs. Results showed that EGFR mutation significantly increased EV release and altered their size, compared to EVs released by wild-type EGFR cells. In addition to classical EV markers such as CD81, Flotillin- 1, and TSG101, Western blot analysis also detected phosphorylated EGFR (p-EGFR) selectively packaged into EVs from PC9 cells. Gefitinib treatment significantly reduced EV secretion in PC9 cells and led to a marked decrease in p-EGFR incorporation into EVs, indicating that EV biogenesis and compostion are modulated by active EGFR signaling. In conclusion, this study highlights the significant influence of EGFR activation on EV secretion and cargo composition while demonstrating that EGFR inhibition via gefitinib alters EV-mediated signaling in lung cancer cells. These findings provide insights into tumor behavior, EV-mediated oncogenic communication, and the potential use of EVs as biomarkers and therapeutic targets in NSCLC.

## Introduction

Lung cancer remain one of the most common causes of cancer-related mortality in worldwide, with non-small cell lung cancer (NSCLC) accounting for approximately 85% of cases [1]. Among the various subtypes of NSCLC, mutations in the epidermal growth factor receptor (EGFR) gene play a crucial role in lung tumorigenesis, making EGFR a primary therapeutic target [2]. Emerging evidence suggests that EGFR signaling not only drives tumor progression, but also influences the release and cargo composition of extracellular vesicles (EVs), which play a key role in intercellular communication within the tumor microenvironment [3].

EVs are heterogeneous lipid bilayer nano-vesicles released into the extracellular space by almost all cells in the body in both physiological and pathophysiological conditions [4]. They are able to facilitate intracellular communication by transporting various bioactive molecules, such as proteins, lipids, nucleic acids (including DNA, RNA, and microRNAs), and other metabolites and signaling molecules that reflect their cell of origin [5]. Upon secretion, EVs can expedite both paracrine and endocrine signaling by transporting their cargo to specific target cells, thereby regulating the activity and behavior of the recipient cells [6].

Depending on their size and biogenesis pathway, EVs are classified into three main groups. Exosomes (also referred to as small EVs, sEVs) have a size range of 30–200 nm and are constitutively produced through the budding of late endosomes known as multivesicular bodies (MVB) containing intraluminal vesicles (ILVs) [7]. When the MVB merges with the plasma membrane, it releases exosomes into the extracellular microenvironment [8]. Microvesicles are bigger in size ranging from 100 nm to 1 µm and are formed directly through budding from the plasma membrane in the process of ectocytosis [9]. Apoptotic bodies are less studied EVs range from 50 to 5000 nm in diameter and are formed during plasma membrane blebbing or when the cells undergo programmed cell death during apoptosis [10].

During cancer progression, cancer cells release an increased amount of EVs, with significant changes in their cargo. These alternations in their composition enhance communication within the tumor microenvironment, promoting metastasis to distant organs or stimulating immune responses [4, 11]. However, the precise mechanism underlying EV biogenesis and its subsequent effect on recipient cells is still not fully understood. Given the critical role of EVs in cellular communication and cancer progression, it is essential to understand the signaling pathways that influence their biogenesis and release. One such pathway is the EGFR signaling pathway, which is frequently dysregulated in various cancers, including lung cancer.

Dysregulation of EGFR signaling, either through receptor overexpression, constitute activation of the receptor due to gene mutation or ligand stimulation, is frequently observed in NSCLC and is associated with poor progression and resistance to therapy [12, 13]. EGFR activating mutations (exon 19 deletion and L858R point mutation) are present in 10–20% of Caucasian patients and in 50% of Asian patients. The mechanism by which EGFR influences tumor behavior and intercellular communication through the modulation of EV biogenesis and alteration of their cargo is not fully understood.

Previous studies have reported that the formation of MVBs during biogenesis of EVs can be stimulated by growth factors as the cells modulate its production of EVs based on its requirements [14]. A recently published review article by Ferlizza et al. [3] demonstrated that EGFR pathway has role in the biogenesis and function of EVs. EGFR can modulate the composition and release of EVs, thereby affecting their ability to mediate communication between cancer cells and the surrounding stromal cells. However, low levels of phosphorylation lead to EGFR undergoing Clathrin-mediated endocytosis and recycling. Conversely, high levels of phosphorylation result in EGFR being ubiquitinated and recognized by the ESCRT complex, which then directs phosphorylated EGFR (p-EGFR) to intraluminal vesicles (ILVs) and potentially to lysosomes for degradation. Furthermore, EGFR mutations may lead to increased auto-phosphorylation and MAPK signaling in certain cancers, such as NSCLC, displaying abnormal ubiquitination [3, 15].

Since the luminal cargo of EVs reflects the cellular context of origin and contains bioactive molecules that are actively expressed during the process of packaging and release [16], EVs released upon EGFR activation in lung cancer may carry mutated forms of EGFR, oncogenic proteins, various types of nucleic acids, and other signaling molecules.

Previous studies reported that p-EGFR and other receptor tyrosine kinases can be detected in EVs purified from the plasma of tumor-bearing mice and from the conditioned media of cultured cancer cells [17]. However, the mechanisms by which mutant EGFR in lung cancer affect the biogenesis and cargo composition of EVs have not been thoroughly investigated. Understanding the exact effects of EGFR activation on the features and functions of EVs is crucial for elucidating how tumors grow and identifying potential therapeutic targets. Given that EV biogenesis and secretion are influenced by EGFR signaling, studying their relationship may provide new insights into tumor biology and immune modulation.

This study aims to isolate and characterize EVs released by PC9 lung cancer cells (harboring an EGFR deletion mutation in exon 19 (2235-2257C)) and A549 lung cancer cells (wild-type EGFR). It further investigates p-EGFR expression in both cells and their secreted EVs and evaluates how gefitinib treatment affects p-EGFR levels in these fractions.

## Materials and methods

### Cell culture

In this study, we used two human lung adenocarcinoma cell lines: PC9 and A549. Both adherent growing cell lines display epithelial morphology, but while PC9 cells harbor an EGFR exon 19 deletion mutation, A549 harbor wild-type EGFR. PC9 cells were cultured in RPMI- 1640 medium (Thermo Fisher Scientific, Waltham, MA, USA) supplemented with 10% fetal calf serum (FCS), 100 U/mL penicillin, and 100 μg/mL streptomycin. A549 cells were cultured in DMEM medium (Sigma-Aldrich, St. Louis, MO, USA) supplemented with 10% FCS and 100 U/mL of penicillin, and 100 μg/mL of streptomycin. Both cell lines were kept at 37 °C with 5% CO₂. Subculturing was performed when cells reached 80–90% confluency using 0.25% trypsin–EDTA to detach the cells, ensuring their continuous proliferation and viability. To evaluate the effect of EGFR inhibition on EV release and p-EGFR expression in both cells and their secreted EVs, PC9 and A549 cells were treated with 200 nM gefitinib for 24 h. Control cells were cultured under identical conditions without gefitinib.

### Isolation of EVs

**Fig. 1:**
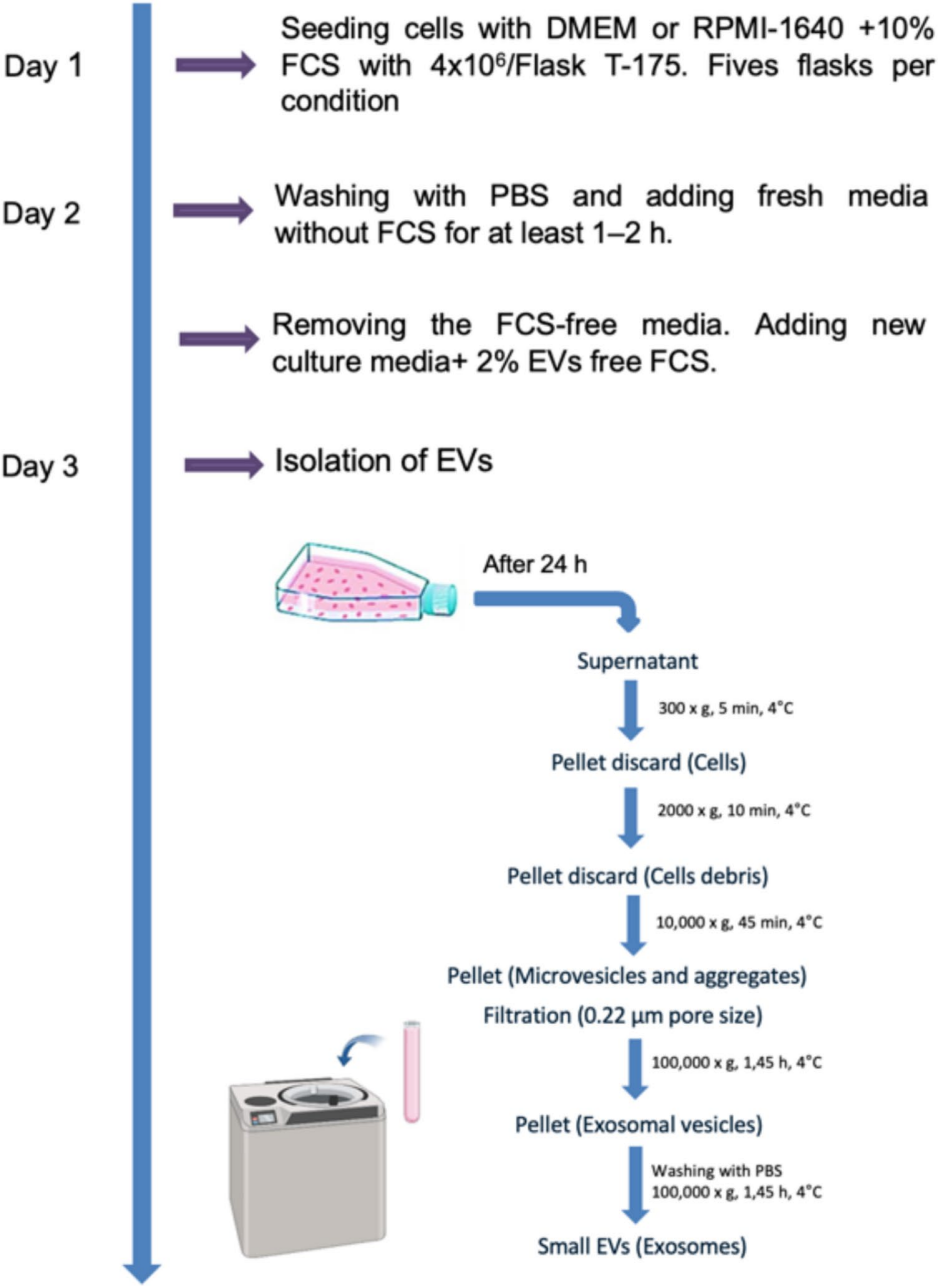
Isolation of EVs by ultracentrifugation. The isolation of EVs by ultracentrifugation involves several key steps. First, cell culture supernatant is collected and subjected to a series of centrifugation steps to remove cellular debris and large particles. Next, the clarified supernatant undergoes ultracentrifugation at high speeds, to pellet EVs. The pellet containing EVs is then carefully resuspended and washed with buffer to further remove contaminants. Finally, the isolated EVs can be resuspended in an appropriate buffer or media without FCS for downstream analyses, such as characterization or functional studies

For isolation of EVs, five T- 175 flasks with 4 × 10^6^ cells each were seeded for each experiment and cell line to obtain sufficient number of EVs. When the cells reached 90% confluency, they were washed twice with PBS and incubated for 24 h with medium containing 2% EV-depleted FCS. Conditioned media (CCM) was collected from both lung cancer cell lines and EVs were isolated using differential centrifugation as previously described, with slight modifications [18]. Briefly, as shown in Fig. 1, 20 mL of CCM from each flask was subjected to centrifugation at 300 × g for 5 min, 2,000 × g for 10 min, and 10,000 × g for 45 min at 4 °C in order to completely eliminate cellular debris and large vesicles. Subsequently, the resulting supernatant was then filtered through a 0.22 μm polyethersulfone filter (Nalgene, Rochester, NY) to eliminate particles exceeding a diameter of 220 nm. To isolate small EVs from the pre-cleared CCM, the filtered supernatant was transferred into ultracentrifuge tubes (Beckman Coulter, Krefeld, Germany) and subjected to high-speed ultracentrifugation at a force of 100,000 xg for a duration of 1:45 h at 4 °C (Optima XPN- 100, Beckman Coulter, Krefeld, Germany) equipped with an SW32 Ti Swinging Bucket rotor (k-factor: 256.8). The resulting EV-enriched pellet was reconstituted in 30 mL of cold PBS, then subjected to another round of ultracentrifugation at 100,000 xg for an additional 1:45 h at 4 °C. The final pellets, containing EVs, were resuspended in 100 µl of PBS and stored at − 80 °C for later use.

### Transmission electron microscopy (TEM)

Electron microscopy was used to confirm and analyze the size and shape of EV. To achieve this, 3 μL of the sample was adsorbed for 5 min onto glow discharged copper mesh grids coated with pioloform carbon. Two drops of distilled deionized water were then used to wash the grids, and then, 2X 4 μl drops of 0.5% aqueous uranyl acetate were added. Before being examined via TEM, the grids were allowed to air dry after any excess stain was removed with filter paper. The JEOL JEM 1400 TEM was used to view the samples at an accelerating voltage of 80 kV. Megaview III digital cameras and iTEM software were used to take the pictures.

#### Nanoparticle tracking analysis (NTA)

Nanoparticle tracking analysis (NTA) quantified the size distribution and concentration of the EVs as reported previously. [19] Briefly, EV samples were diluted in HBSS buffer to a final volume of 1 mL (dilution range: 1:50–1:2000). The optimal measurement concentrations were determined by testing the particle count per frame within the range of 140 to 200 particles per frame. The manufacturer’s default software settings were used for EVs. Two cycles were conducted for each measurement, involving the scanning of 11 cell positions and capturing 30 frames per position. The measurements were performed under the specified settings. Focus: Autofocus; Camera sensitivity for all samples: 79; Shutter speed: 70; Scattering intensity: Automatically detected; Cell temperature: 24 °C. Following capture, the videos were subjected to specific analysis parameters using the built-in ZetaView Software 8.05.11 SP1: Minimum area: 5, minimum brightness: 30, maximum area: 1000. Hardware: CMOS camera; 40 mW embedded laser at 488 nm.

#### Western blotting

PC9 and A549 were lysed with 1 × RIPA buffer (50 mM Tris–HCl, pH 7.6, 150 mM NaCl, 1% Triton- 100, 0.5% sodium deoxycholate, 0.1% SDS, protease and phosphatase cocktail inhibitor). The cell lysate was centrifuged at 10,000 × g for 10 min at 4 °C. The protein concentrations of the supernatant were then estimated using BCA protein assay kit (Thermo Fisher Scientific Inc, Cat#: 23,227). The EVs isolated from PC9 and A549 CCM were lysed with 1 × RIPA buffer. Then, equal volumes of protein (20 μg for EVs and 10 μg for cell lysates) for each sample were prepared and separated by 8–12% SDS-PAGE gel and then transferred to polyvinylidene fluoride (PVDF) membrane or 0.45 µm nitrocellulose membranes (Sigma-Aldrich, Massachusetts, USA, GE10600003). Subsequently, the membrane was washed with TBS four times for 5 min each at room temperature and stained with Ponceau S to visualize the protein bands followed by blocking with 5% BSA in TBS-T for an hour at room temperature to prevent non-specific binding. After being rinsed four times for 5 min each time with TBS buffer, the membranes were incubated with primary antibodies of interest (Table 1) overnight at 4 °C. Membranes were washed again four times for 5 min with TBS-T, and HRP-conjugated secondary anti-mouse IgG or anti-rabbit IgG were added for 1 h at room temperature. Membranes were again washed four times, and protein bands were visualized using an enhanced chemiluminescence (ECL) substrate (GE Healthcare Life Science) and analyzed using an Odyssey Fc Imager (LI-COR Biosciences).

**Table 1:** Primary and secondary antibodies

Antibody	Dilution	Ca#	Source
CD81	1:1000	166,029	Santa cruz biotechnology, Dallas, TX, USA
Flotillin- 1	1:5000	610,821	BD Biosciences,
TSG101	1:5000	7964	Santa cruz biotechnology, Dallas, TX, USA
β-Action	1:5000	47,778	Santa cruz biotechnology, Dallas, TX, USA
EGFR	1:1000	4267	Cell signaling, Danvers, MA, USA
p-EGFR^Tyr1068^	1:1000	2234	Cell signaling, Danvers, MA, USA
AKT	1:1000	9272	Cell signaling, Danvers, MA, USA
p-AKT^ser473^	1:1000	9271	Cell signaling, Danvers, MA, USA
p44/42 MAPK (ERK1/2)	1:1000	9102	Cell signaling, Danvers, MA, USA
P-p44/42 MAPK (ERK1/2) _Thr202/Tyr204_	1:1000	4370	Cell signaling, Danvers, MA, USA
Calnexin	1:1000	2433	Cell signaling, Danvers, MA, USA
Anti-mouse HRP	1:5,000	7076	Cell signaling, Danvers, MA, USA
Anti-Rabbit HRP	1:5,000	7074	Cell signaling, Danvers, MA, USA

#### Statistical analysis

The software SPSS V22.0 (IBM, USA) was used for all statistical analyses, and GraphPad Prism10.1.2. (GraphPad Software, San Diego, CA, USA) was used for producing the graphs. One-way ANOVA Student’s t test was used to analyze the differences’ statistical significance. The threshold of *P* < 0.05 was considered statistically significant.

## Results

### Analysis of EGFR activation and its downstream signaling

**Fig. 2:**
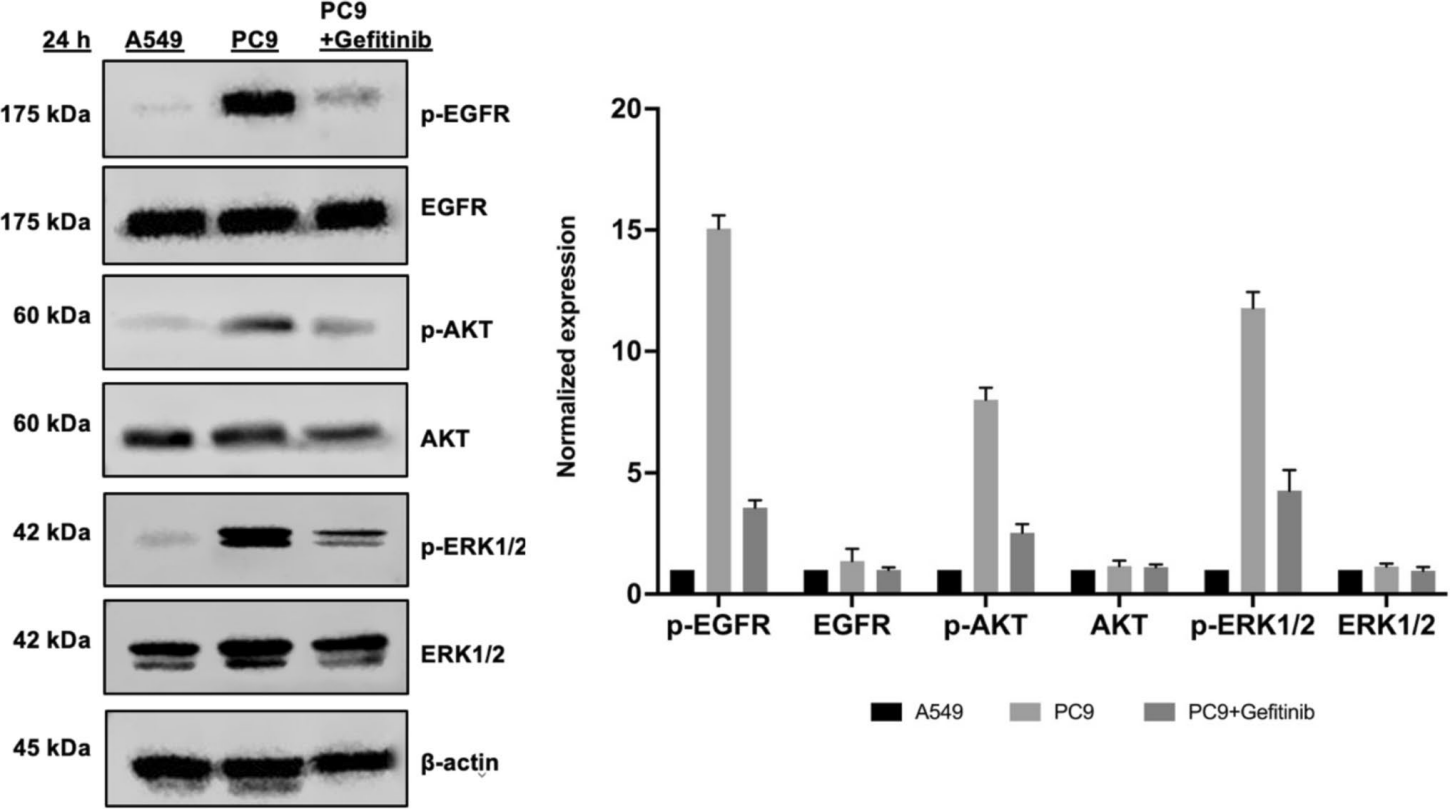
Detection of the activation status of EGFR signaling in A549 and PC9 cells by Western blot analysis. Immunoblots were incubated with EGFR, p-EGFR, AKT, p-AKT, ERK1/2 and p-ERK1/2. Phosphorylation of p-EGFR, p-AKT, and p-ERK1/2 was detected in PC9 cells (middle blot) but not in A549 cells (left blot). PC9 cells treated with 200 nM gefitinib for 24 h significantly inhibited p-EGFR and its downstream signaling (right blot). Expression levels normalized to total β-actin are depicted in the right graphs. All data represent mean ± SD of three independent experiments

To investigate the activation status of EGFR and its downstream signaling pathways, we performed a comparative analysis between PC9 and A549 cancer cell lines. PC9 cells harbor activating mutations in EGFR, while A549 cells are wild-type for EGFR, providing a useful model to study differential EGFR signaling. Western blot analysis was used to detect the endogenous EGFR and p-EGFR, which indicates its active state. As illustrated in Fig. 2, EGFR was expressed in both cell lines, while p-EGFR was readily detected in PC9 cells but was absent in A549 cells, confirming the constitutive activation of EGFR in PC9 cells. Furthermore, the western blot results indicated that this sustained EGFR activation in PC9 cells triggers downstream signaling pathways, specifically the phosphorylation of AKT (p-AKT) and phosphorylation of ERK1/2 (p-ERK1/2), key components of cell survival and proliferation, whereas these pathways were inactive in A549 cells. However, the cell treated with 200 nM gefitinib for 24 h significantly inhibited p-EGFR and its downstream signaling.

### Characterization of EVs derived from EGFR-mutant and EGFRwt cells

**Fig. 3:**
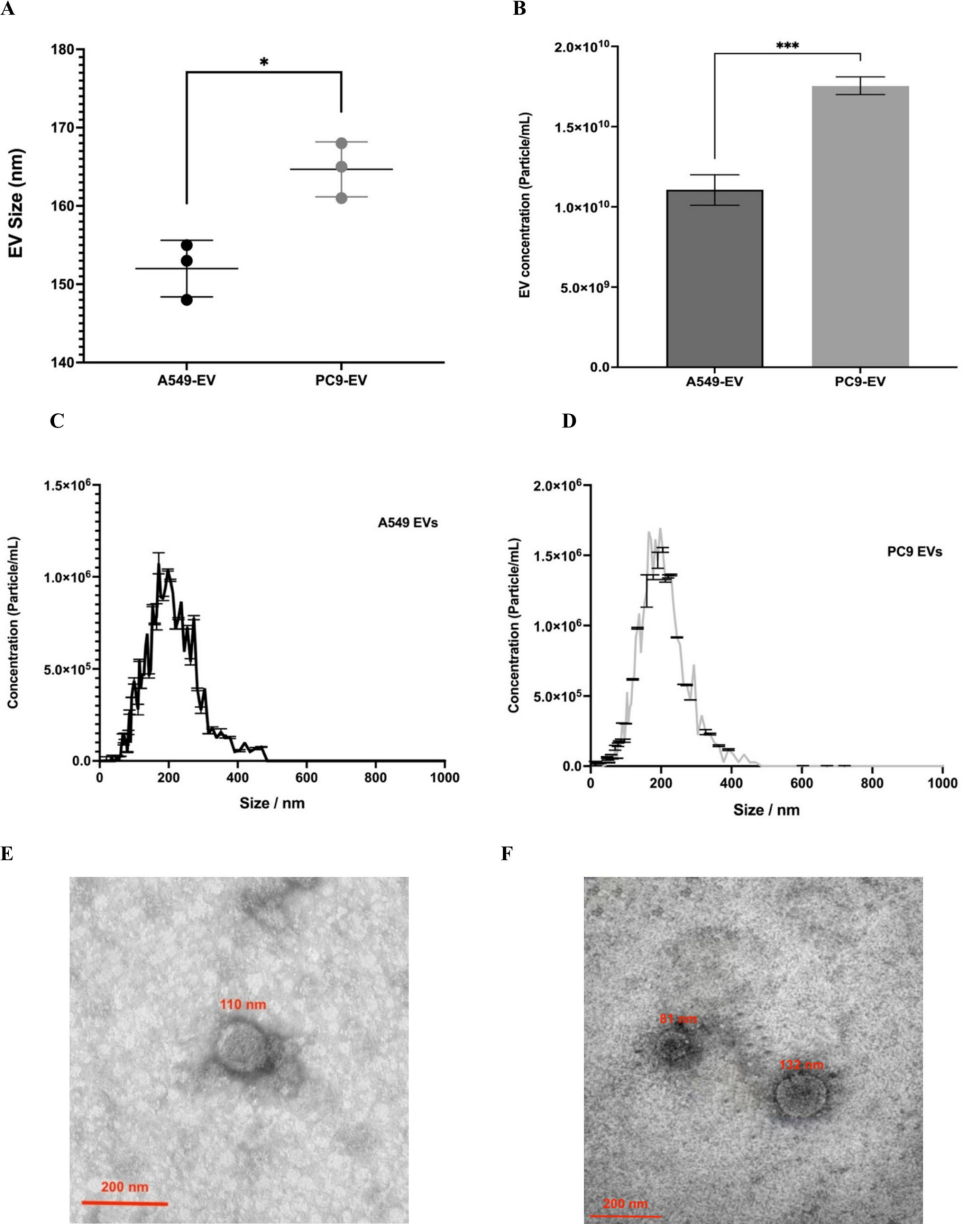
Isolation and characterization of EVs from lung cancer cell lines. **A**. NTA results show the median size of EVs from both PC9 and A549 cell lines from three independent EV isolations. T test analysis reveals a significantly bigger size of PC9-EVs compared to A549. *P* ≤ 0.02. **B**. Quantitative analysis reveals a significantly higher concentration of EVs from EGFR-mutant PC9 cells compared to EGFRwt A549 cells. The size distribution of the isolated EVs from A549 **C** and PC9 **D** predominantly ranges between 30 and 200 nm, consistent with typical EV sizes. TEM analyses revealed a majority of EVs from A549 **E** and PC9 **F** are cup-shaped morphology ranging from70 to 150 nm in size. C-F representative data from one of three independent experiments.

To confirm the isolation and purity of EVs derived from PC9 and A549 cell lines, NTA, TEM and Western blotting were performed. The NTA results showed that the isolated EVs had a size range between 30 to 200 nm, with a median size of 165 nm (modal: 161 nm) (PC9) and 153 nm (modal: 148 nm) (A549), respectively, (Fig. 3A). Quantitative analysis revealed that EGFR-mutant PC9 cells released a significantly higher number of EVs (1.75E + 10 particles/mL) compared to EGFRwt A549 cells (1.10E + 10 particles/mL) (*p* = 0.001) (Fig. 3B, D). This confirms that the constitutive activation of EGFR in PC9 cells enhances EV secretion, supporting the role of EGFR signaling in regulating EV biogenesis and size. TEM analyses verifies a vesicle size of approximately 70–150 nm size and shows the typical cup-shaped morphology  of small EVs, for both, A549 (Fig. 3E) and PC9 cells derived EVs (Fig. 3F).

###  Detection of EV markers confirm enrichment and purity of EVs 

**Fig. 4:**
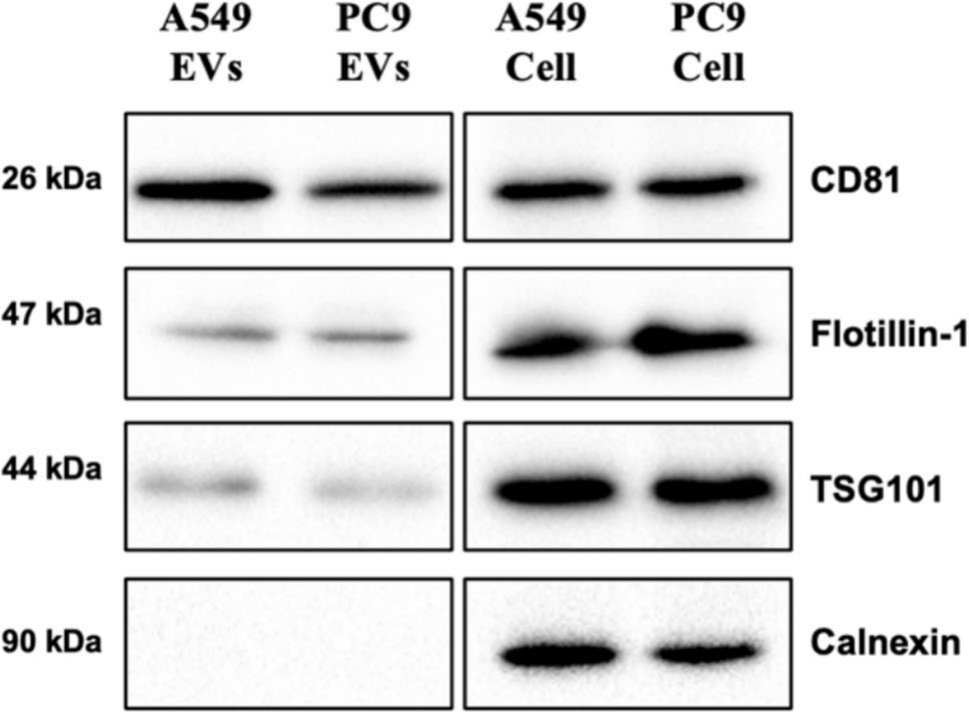
Identification of EV markers in isolated EVs. Western blot analysis of EVs derived from A549 and PC9 cells. The EV markers CD81, Flotillin- 1, and TSG101 were detected in EV samples (left blot), confirming the presence of EV-associated proteins. Calnexin, an endoplasmic reticulum (ER) marker, was absent in the EV samples but present in whole-cell lysates, indicating the successful isolation of pure EVs without significant cellular contamination

To validate the successful isolation of EVs, we performed Western blot analysis to detect a panel of EV markers in the EV samples. The EV markers CD81, Flotillin- 1, and TSG101 were detected in EVs derived from both A549 and PC9 cells, confirming the presence of EV-associated proteins (Fig. 4). Additionally, these markers were also observed in whole-cell lysates, further supporting their expression in the originating cells. Importantly, Calnexin, an endoplasmic reticulum (ER) marker, was absent in the EV samples but present in whole-cell lysates, confirming the purity of the isolated EVs and the absence of significant cellular contamination.

### p-EGFR is packaged into EVs

**Fig. 5:**
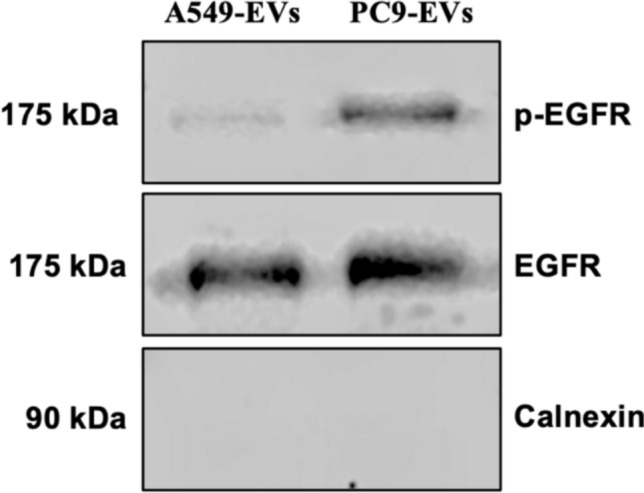
p-EGFR is selectively packaged into EVs from PC9 cells. Western blot analysis of EVs isolated from A549 (EGFRwt) and PC9 (EGFR-mutant) cells. p-EGFR was prominently detected in EVs from PC9 cells (right blot), whereas it was undetectable in A549-derived EVs (left blot), consistent with the constitutive activation of EGFR in PC9 cells. Total EGFR was present in EVs from both cell lines, confirming the incorporation of EGFR in EVs. Calnexin was absent in all EV samples, indicating the purity of the isolated EVs

To investigate whether EGFR and its phosphorylated form (p-EGFR) are incorporated into EVs, we performed Western blot analysis on EVs isolated from PC9 and A549 cells. The results showed that p-EGFR was prominently detected in EVs derived from PC9 cells, whereas it was absent in EVs from A549 cells. This finding is consistent with the constitutive activation of EGFR in PC9 cells, which harbor an EGFR mutation leading to continuous phosphorylation. In contrast, A549 cells, which express wild-type EGFR, do not exhibit detectable levels of phosphorylated EGFR in their EVs. Additionally, the presence of total EGFR was confirmed in EVs from both cell lines, further supporting the incorporation of EGFR-related signaling molecules into EVs. The absence of Calnexin, an ER marker, in EV samples indicates that the observed signals are not due to cellular contamination, confirming the purity of the isolated EVs (Fig. 5).

### Gefitinib treatment impacts EVs release 

**Fig. 6:**
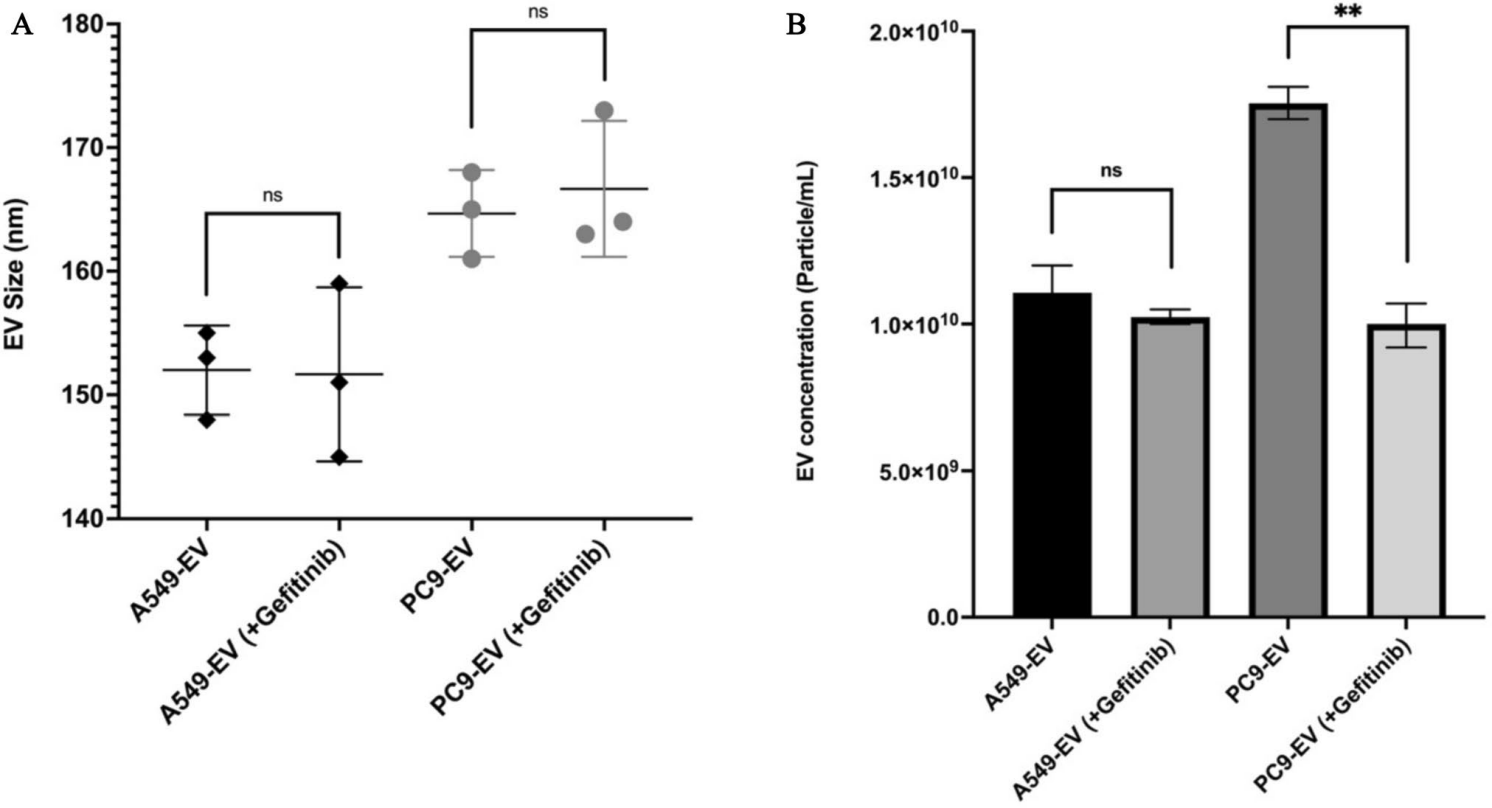
Effect of gefitinib treatment on EV size and secretion. **A** NTA showing the size distribution of EVs derived from A549 and PC9 cells, both with and without 20 nM gefitinib treatment for 24 h. No significant differences were observed in EV size across all conditions. **B** Gefitinib treatment significantly reduced the number of EVs secreted by PC9 cells (~ 47% decrease, *p* < 0.01), whereas it had no significant effect on A549-derived EVs. Error bars represent mean ± standard deviation (SD). Statistical significance: ns = not significant, ** *p* < 0.01

To evaluate the impact of EGFR inhibition on EV secretion, both PC9 and A549 cells were treated with 20 nM gefitinib for 24 h, and the number of released EVs were quantified using NTA. The results demonstrated that gefitinib treatment significantly reduced the number of EVs secreted by PC9 cells, while having no significant effect on A549 cells (Fig. 6). Specifically, PC9-derived EVs decreased from (1.75E + 10 particles/mL) in untreated cells to (0.92E + 10 particles/mL) following gefitinib treatment with statistical significance (*p* = 0.01), indicating an approximate 47% reduction in EV secretion. In contrast, A549-derived EV secretion remained relatively unchanged, with EV numbers measuring (1.10E + 10 particles/mL) before treatment and (1.05E + 10 particles/mL) after treatment, suggesting that gefitinib’s effect on EV secretion is specific to EGFR-mutant cells.

### Reduced p-EGFR expression in EVs after gefitinib-treatment

**Fig. 7:**
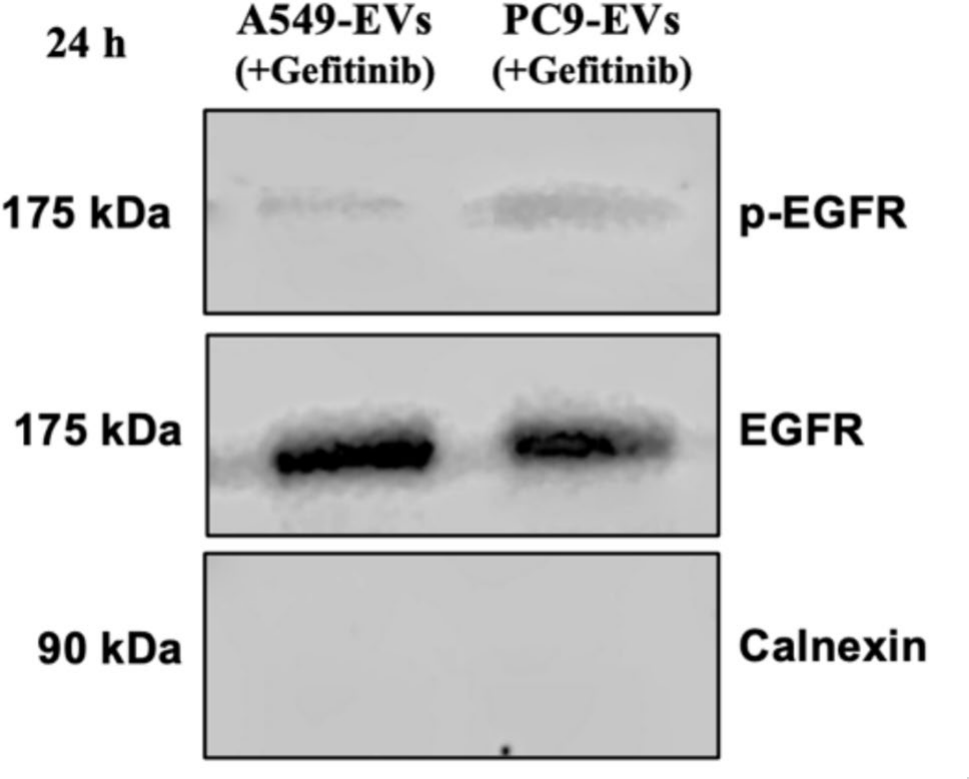
Gefitinib inhibits p-EGFR expression in EVs. Western blot analysis of EVs isolated from A549 and PC9 cells, both treated with 200 μM gefitinib for 24 h. p-EGFR levels were significantly reduced in gefitinib-treated PC9-derived EVs, confirming the inhibition of EGFR signaling. Total EGFR was present in EVs from both cell lines, indicating its incorporation into EVs independent of phosphorylation. Calnexin was absent in all EV samples, confirming the purity of the isolated EVs and ruling out cellular contamination

To assess the effect of EGFR kinase inhibition on p-EGFR levels in EVs, EVs were isolated from PC9 and A549 cells after treatment with 200 μM gefitinib for 24 h, followed by Western blot analysis. The results demonstrated that p-EGFR levels were significantly reduced in EVs derived from gefitinib-treated PC9 cells, confirming the effective inhibition of EGFR signaling in EVs (Fig. 7). In contrast, EVs from A549 cells exhibited minimal p-EGFR expression, and gefitinib treatment had no significant effect, consistent with their wild-type EGFR status. Importantly, total EGFR was present in EVs from both cell lines, indicating that EGFR incorporation into EVs is independent of its phosphorylation status. Additionally, the absence of Calnexin, an ER marker, in all EV samples confirms the purity of the isolated EVs and rules out contamination from cellular debris.

## Discussion

The EGFR signaling pathway is well documented for its pivotal involvement in various cellular processes. EGFR mutations are present in 10–20% of Caucasian and 50% of Asian NSCL patients, leading to constitutive activation of downstream pathways. This dysregulation leads to poor prognosis and resistance to therapy [2]. However, its role in regulating EVs is not clear. The present study aimed to better understand how constitutive activation of EGFR influences the release of EVs and their miRNA cargo composition, which could offer insight into tumor progression and potential therapeutic targets.

In recent years, there has been a growing interest in understanding intercellular communication facilitated by EVs. These are the carriers that transport various types of proteins, lipids, nucleic acids, and signaling molecules. Among these vesicles, small EVs (exosomes) and large EVs (microvesicles) have attracted a lot of attention as they are found in biological fluids and can facilitate intercellular communication and initiate signaling cascades [20].

Our findings demonstrated that constitutive EGFR activation affects EV secretion in lung cancer cells. Specifically, the PC9 cell line harboring mutant EGFR showed a significantly increased EV release compared to A549 harboring wild-type EGFR. This difference was no longer observed after treatment with the EGFR kinase inhibitor gefitinib, which significantly reduced the release of EVs from PC9 but not A549. These results suggest that EGFR signaling positively regulates EVs secretion. These results are consistent with previous studies that suggested EGFR signaling may affect EV biogenesis and release. Montermini et al. [21] detected p-EGFR and several other tyrosine kinases in EVs purified from the plasma of tumor-bearing mice and from the conditioned media of cultured cancer cells. Interestingly, she showed that inhibition of oncogenic EGFR with the second generation of  tyrosine kinase inhibitors (TKI), including CI- 1033 and PF- 00299804, triggers the release of exosome-like extracellular vesicles and affects the level of their phosphoprotein and DNA content.

Also, Zhou et al. [22] showed that EGF-activated cells and subsequent EGFR signaling led to a decrease in exosome production accompanied by a promotion of wound healing in BUMPT cells. Conversely, inhibition of EGFR by gefitinib increased exosome production, accompanied by inhibition of wound healing. This decrease in EV release upon EGFR activation may be due to the process of EGFR ubiquitination, in which activated EGFR undergo rapid Clathrin-mediated endocytosis and are recognized by the ESCRT complexes, where the lysosomally targeted receptors, such as activated EGFR, are sorted onto the ILVs of MVBs for subsequent lysosomal degradation or undergo recycling by being retained on the limiting membrane of the MVB and returned to the plasma membrane and released as exosomes [23, 24].

In our study, NTA revealed that the size of EVs derived from both cell lines ranged between 30 and 200 nm, confirming successful EV isolation. It also showed that mutated EGFR cells produced EVs that were bigger in size compared to EVs derived from wild-type EGFR cells. This size increase could be due to alterations in the cellular machinery involved in EV biogenesis, influenced by EGFR signaling. However, gefitinib treatment did not significantly alter the size of EVs in either PC9 or A549 cells. This suggests that size regulation of EVs is most likely independent of EGFR phosphorylation status or that longer inhibition periods may be required to observe measurable changes. Previous studies have indicated that tumor-derived EVs display heterogeneity in size, which could be influenced by biogenesis pathways, lipid composition, or cargo loading [25].

Furthermore, the presence of EV-specific proteins such as CD81, TSG101, and Flotillin- 1 in the isolated EVs from both cell lines confirmed their identity and the successful isolation process. These proteins are well-established markers for EVs and are commonly used to validate their presence and purity [26]. In addition to classical EV markers, immunoblotting shows that EVs released by mutated EGFR cell line PC9 but not wild-type EGFR cell line A549 carry p-EGFR, indicating that the phosphorylated form of EGFR is successfully packaged into EVs. Importantly, gefitinib treatment significantly inhibited the phosphorylation in PC9 cells and led to reduced p-EGFR levels in PC9-derived EVs, confirming that p-EGFR incorporation into EVs is dependent on active EGFR signaling. Western blot analysis showed that while total EGFR remained detectable in EVs from both treated and untreated conditions, the enrichment of phosphorylated EGFR was disrupted following gefitinib treatment. This suggests that p-EGFR sorting into EVs may involve ESCRT-dependent pathways, which regulate cargo selection [24]. Given the role of EVs in transferring oncogenic signals, reducing p-EGFR packaging into EVs may limit the ability of tumor-derived EVs to propagate EGFR-driven signaling, potentially contributing to the therapeutic effects of TKIs.

Our findings from this study contribute to the growing body of evidence that EVs have a significant impact on cancer biology, specifically in relation to EGFR signaling. Previous studies have proven that EVs can carry oncogenic proteins, RNA, and other bioactive molecules, facilitating communication within the tumor microenvironment and promoting metastatic processes. [20]. Furthermore, our results demonstrated that gefitinib significantly reduced the secretion of EVs from PC9 cells, whereas it had no significant effect on A549-derived EVs. This suggests that EGFR activation impacts EV biogenesis leading to an increased EV release.. In addition, Lin et al. [27] demonstrated that EVs derived from EGFR-mutant NSCLC cells increased the sensitivity of recipient cells to the tyrosine kinase inhibitor gefitinib. This suggests that these EVs may play a role in modulating drug response by enhancing the efficacy of targeted therapies in EGFR-mutant NSCLC.

However, the study also raises several questions that need further investigation. We still need to fully elucidate the exact mechanism by which EGFR signaling modulates EV biogenesis and specific cargo selection. Future research should explore how gefitinib treatment affects the functional role of EVs in tumor progression. In particular, investigating the ability of EVs from gefitinib-treated PC9 cells to influence recipient cell behavior could provide deeper insights into their role in drug resistance mechanisms. Understanding these mechanisms could provide insights into the development of targeted therapies that disrupt EV-mediated communication in the tumor microenvironment. Furthermore, we need to explore in more detail the functional implications of altered EV composition on recipient cells and their role in tumor progression.

In conclusion, our study underlines the significant impact of EGFR signaling on EV secretion and cargo composition in lung cancer cells. By demonstrating that gefitinib reduces EV secretion and p-EGFR incorporation in EVs, our findings highlight a potential mechanism through which EGFR inhibitors may suppress oncogenic intercellular signaling. The findings establish a basis for additional investigation into the role of EVs in cancer progression and their potential as therapeutic targets and biomarkers. Understanding the interplay between EGFR signaling and EVs could open new opportunities for developing innovative strategies to fight lung cancer.

## Data Availability

No datasets were generated or analyzed during the current study.
